# Phylogenetic analysis of the Neotropical Albitarsis Complex based on mitogenome data

**DOI:** 10.1186/s13071-021-05090-w

**Published:** 2021-11-27

**Authors:** Brian P. Bourke, Silvia A. Justi, Laura Caicedo-Quiroga, David B. Pecor, Richard C. Wilkerson, Yvonne-Marie Linton

**Affiliations:** 1 Walter Reed Biosystematics Unit, Smithsonian Institution Museum Support Center, MRC-534, 4210 Silver Hill Rd., Suitland, MD 20746 USA; 2 Walter Reed Army Institute of Research, 503 Robert Grant Avenue, Silver Spring, MD 20910 USA; 3 Department of Entomology, Smithsonian Institution-National Museum of Natural History, 10th St NE & Constitution Ave NE, Washington, DC 20002 USA

**Keywords:** Malaria, Mitogenome, Mosquito, Phylogenetics, *Plasmodium*, Vector

## Abstract

**Background:**

Some of the most important malaria vectors in South America belong to the Albitarsis Complex (Culicidae; Anophelinae; *Anopheles*). Understanding the origin, nature, and geographical distribution of species diversity in this important complex has important implications for vector incrimination, control, and management, and for modelling future responses to climate change, deforestation, and human population expansion. This study attempts to further explore species diversity and evolutionary history in the Albitarsis Complex by undertaking a characterization and phylogenetic analysis of the mitogenome of all 10 putative taxa in the Albitarsis Complex.

**Methods:**

Mitogenome assembly and annotation allowed for feature comparison among Albitarsis Complex and *Anopheles* species. Selection analysis was conducted across all 13 protein-coding genes. Maximum likelihood and Bayesian inference methods were used to construct gene and species trees, respectively. Bayesian methods were also used to jointly estimate species delimitation and species trees.

**Results:**

Gene composition and order were conserved across species within the complex. Unique signatures of positive selection were detected in two species—*Anopheles janconnae* and *An. albitarsis* G—which may have played a role in the recent and rapid diversification of the complex. The *COI* gene phylogeny does not fully recover the mitogenome phylogeny, and a multispecies coalescent-based phylogeny shows that considerable uncertainty exists through much of the mitogenome species tree. The origin of divergence in the complex dates to the Pliocene/Pleistocene boundary, and divergence within the distinct northern South American clade is estimated at approximately 1 million years ago. Neither the phylogenetic trees nor the delimitation approach rejected the 10-species hypothesis, although the analyses could not exclude the possibility that four putative species with scant a priori support (*An. albitarsis* G, *An. albitarsis* H, *An. albitarsis* I, and *An. albitarsis* J), represent population-level, rather than species-level, splits.

**Conclusion:**

The lack of resolution in much of the species tree and the limitations of the delimitation analysis warrant future studies on the complex using genome-wide data and the inclusion of additional specimens, particularly from two putative species, *An. albitarsis* I and *An. albitarsis* J.

**Graphical Abstract:**

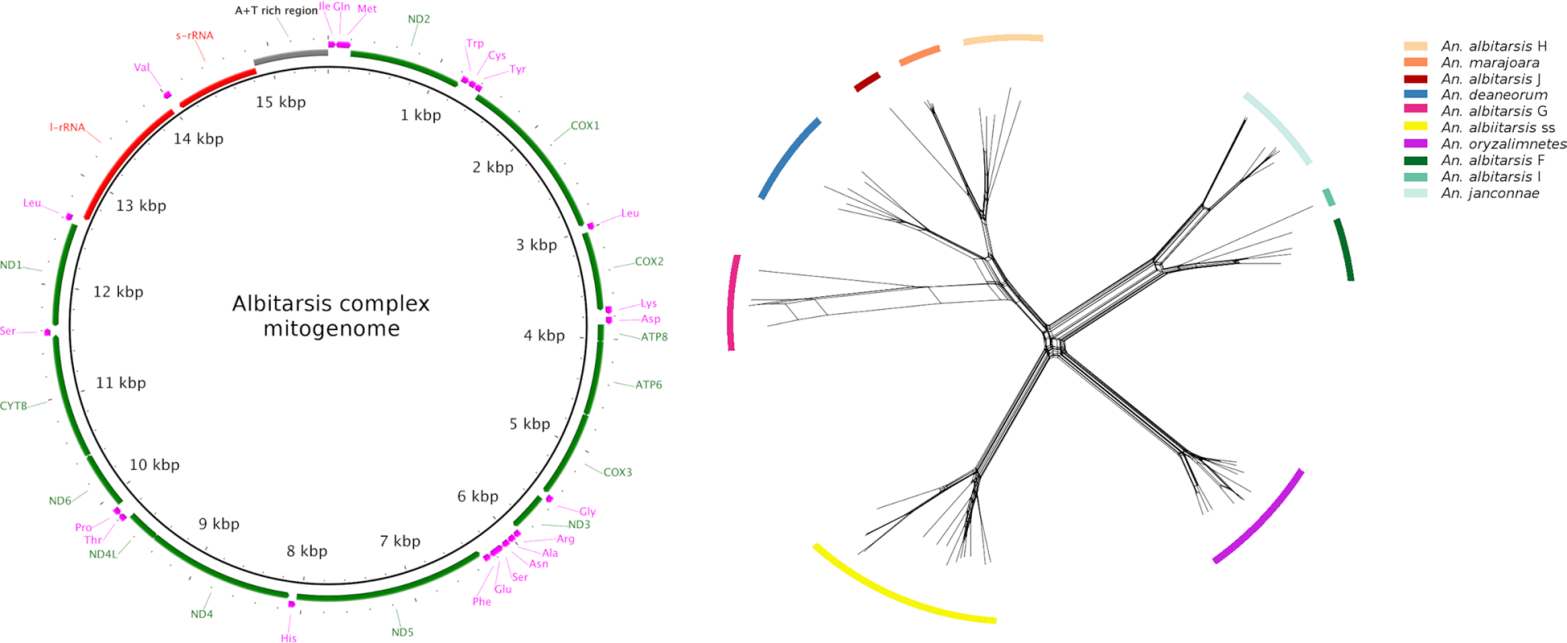

**Supplementary Information:**

The online version contains supplementary material available at 10.1186/s13071-021-05090-w.

## Background

Vector-borne diseases account for almost one fifth of the global burden of infectious diseases [1]. The most important of these is malaria, which is responsible for more than 400,000 deaths annually [2]. This disease is caused by *Plasmodium* parasites transmitted to humans through the bites of *Anopheles* mosquitoes. Five species of *Plasmodium* cause malaria in humans [3, 4], but two—*Plasmodium falciparum* and *Plasmodium vivax—*are considered the greatest threat. The former is the predominant species in Africa, where almost 95% of global malaria cases and deaths occur, while the latter predominates in the Americas, accounting for 75% of the approximately 1 million cases reported annually [2].

Within the Americas, *Plasmodium* is transmitted by a diverse array of mosquito vectors [5], the most important of which is *Anopheles darlingi*, found through much of South and Central America [6]. Many of the other dominant vector species [5] are morphologically indistinguishable from benign close relatives, and the existence of such cryptic groups or *complexes* obscures ecological and life history differences that may be of public health importance and complicates incrimination and targeted control strategies. Among the most important of these are species belonging to the Albitarsis Complex [5]. This complex currently comprises 10 known and putative species, variously distributed across Argentina, Bolivia, Brazil, Colombia, French Guiana, Paraguay, Trinidad, and Venezuela [7–11]. At least three are highly competent vectors. The importance of *An. marajoara* in *Plasmodium* transmission in the Brazilian Amazon is comparable to that of *An. darlingi* in established forest settlements [12], while it has been found to be the most important malaria vector at emerging frontier settlements [13]. It is also found as a potentially important vector at artisanal gold mines in the forests of French Guiana [14]. In laboratory-based transmission studies, *Plasmodium* infection rates of *An. deaneorum* were comparable to the primary South American malaria vector, *An. darlingi* [15, 16]. In the field, *An. deaneorum* are found naturally infected with *P. vivax* and/or *P. falciparum*, and natural infection rates combined with high local abundance in the state of Acre, Brazil, support its importance in local malaria transmission [17]. *Anopheles janconnae* [18] is the primary malaria vector in the savannah surrounding Boa Vista in the Brazilian state of Roraima. The public health importance of the remaining members of the Albitarsis Complex is poorly understood, but based on overlap of distribution of sustained malaria transmission, the spatial evolutionary and ecological vicariance analysis (SEEVA) of Foley et al. [19] suggested that *An. albitarsis* G, H, and I may also play significant roles in malaria transmission. Difficulty with species identification can lead to problems with vector incrimination, and surveys of *Plasmodium* vectors frequently identify these specimens only as *An. albitarsis* sensu lato [20–25]. In addition, many of the species in the complex, including *An. marajoara* and *An. deaneorum*, are predicted to expand their distributions due to the effects of climate change and are therefore likely to be of increasing public health importance in the future [26].

A variety of morphological [27–30] and molecular [7, 9, 31–39] approaches have been employed to discriminate species and explore species relationships within the Albitarsis Complex. Phylogenetic studies have played a particularly important role in describing this species complex’s diversity, but they have recovered conflicting topologies and uncertainty concerning species relationships [7, 35–38]. The work of Ruiz-Lopez et al. [7] and Motoki et al. [11] supported a basal split that separates northern and southern continental species at the mitochondrial cytochrome c oxidase I (*COI*) gene. However, this branching order has not been recovered in other studies using other regions of the *COI* gene [36] or the mitogenome [37, 38]. Hypothesized sister relationships, such as those with *An. albitarsis* G and *An. oryzalimnetes*, also vary both within and between these studies.

The mitogenome has rapidly become a popular marker for phylogenetic analyses in recent years. Given its maternal inheritance and limited recombination [40], different regions in the mitogenome are expected to have the same evolutionary history. Variation among mitochondrial gene trees is therefore generally attributed to systematic error rather than biological process [41]. The conserved gene composition of the mitogenome makes establishing orthology simple, and the mitogenome’s high mutation rates and considerable size (reducing stochastic error) make it attractive for establishing phylogenies of closely related species, such as the putative species within the Albitarsis Complex. We then describe mitogenomic relationships among these species using traditional phylogenetic and multispecies coalescent methods and discuss the significance of our findings in the context of Albitarsis Complex history and phylogeography. Our findings provide important insights for future studies of speciation in the complex.

## Methods

### Collection

Specimens sequenced for this molecular study were previously morphologically identified in Ruiz et al. [7] and Motoki et al. [11] as belonging to the Albitarsis Complex using the available keys [30, 42]. These specimens have been stored as vouchers or had their DNA stored in archive collections of the Walter Reed Biosystematics Unit (WRBU), Smithsonian Institution–National Museum of Natural History, Museum Support Center (MSC), Suitland, Maryland, USA, or in the frozen tissue collection at the Natural History Museum, London, UK.

### DNA extraction and sequencing

DNA was extracted according to the method reported by Gilbert et al. [43]. Each specimen was placed in a 1.5 ml tube and fully immersed in a digestion buffer, which consisted of 3 mM CaCl_2_, 2% sodium dodecyl sulphate (SDS), 40 mM dithiothreitol (DTT), 250 µg/ml proteinase K, 100 mM Tris buffer pH 8, and 100 mM NaCl. This was incubated for at least 18 h on a slow shaker at 55 °C. DNA was then precipitated using a solution containing 0.6× digestion buffer volume of isopropanol, 0.1× digestion buffer volume of 3 M sodium acetate pH 5.2, and 0.01× digestion buffer volume of Ambion GlycoBlue. The detailed protocol was published in Justi et al. (2021) and is available on the WRBU website at https://wrbu.si.edu/docs/sops/MolLabSOP1.pdf.

DNA was quantified for all samples using the Qubit High Sensitivity Assay Kit for fluorometric quantification (Thermo Fisher Scientific, Waltham, MA, USA). In cases where the DNA concentration was too low to quantify, the DNA solution was concentrated by evaporation using a Savant SpeedVac Plus centrifuge vacuum concentrator and re-quantified.

Illumina library preparation was performed using KAPA HyperPlus Kits (Roche, Basel, Switzerland). Highly fragmented DNA identified by an Agilent 4200 TapeStation automated electrophoresis system (Agilent Technologies, Santa Clara, CA, USA) required DNA end repair and A-tailing steps following the manufacturer’s protocol. Adapter ligation and polymerase chain reaction (PCR) amplification was in accordance with the manufacturer’s recommendations. Subsequent quality control and fragment distribution were again assessed with the 4200 TapeStation (Agilent Technologies), and AMPure XP bead (Beckman Coulter, Brea, CA, USA) clean-up was performed to remove adapter dimers and other impurities, when necessary. The detailed protocol, based on the KAPA HyperPlus technical datasheet, is available on the WRBU website at https://wrbu.si.edu/docs/sops/MolLabSOP2.pdf. Sequencing was performed using the NovaSeq Illumina platform (PE 2 × 150) at the Walter Reed Army Institute of Research (WRAIR), Silver Spring, MD, USA. Read quality was checked using fastqc [44].

### Mitogenome assembly and annotation

Mitogenomes were assembled de novo from whole-genome data using NOVOPlasty [45]. Assembled mitogenomes were then annotated for protein-coding genes (PCGs) and ribosomal RNAs (rRNAs) in Geneious Prime 2020.1.1 (https://www.geneious.com) using the *An. albitarsis* mitochondrial reference genome (NC_020662), and adjusted, where appropriate, using the recommendations of Cameron [46]. The transfer RNAs (tRNAs) were inspected by comparison with the annotation approach described by Jühling et al. [47] and implemented in MITOS [48]. Annotations in the Albitarsis Complex were visualized using the BLAST Ring Image Generator [49]. Each specimen was identified according to the 10-member Albitarsis Complex detailed in Motoki et al. [11], using *COI* barcode data described therein. Sequencing yielded 35 mitogenomes, which were combined with an additional 25 mitogenomes from GenBank for subsequent mitogenomic analyses (Additional file 1: Table S1).

### Alignments

All gene sequences were aligned by nucleotide position using the MUSCLE [Multiple Sequence Comparison by Log-Expectation] algorithm [48] implemented in SeaView [50]. The PCGs were also aligned by amino acid using TranslatorX [51]. AT composition and site variation was calculated using MEGA [52]. The extent of substitution saturation at the third codon position was tested using the index of substitution saturation (ISS) test of Xia et al. [53] and implemented in DAMBE [54].

### Selection analyses

Radical physicochemical amino acid changes at the 13 PCGs of the mitogenome were identified using TreeSAAP [55]. This approach compares the distribution of physicochemical changes inferred from a phylogenetic tree with the expected distribution of random changes expected under selective neutrality. The magnitude of the changes was assigned to eight classes, representing conservative (tending towards class 1) and radical (tending towards class 8) changes in amino acid characteristics. Only the most radical changes (classes 6 to 8; critical *Z-score* values for *P* = 0.001 of 3.09 and − 3.09, indicating positive and negative selection, respectively) were of interest. To reduce the potential for false-positive results, physicochemical properties that had an accuracy of detection of selection lower than 85% (11 of 31) were excluded from the analysis, as recommended by McClellan and Ellison [56]. Each gene was analyzed separately using a maximum likelihood tree of the PCG alignment as the input tree. TreeSAAP results were summarized and viewed in IMPACT_S [57]. Under the evolutionary pathway (Evpthwy) tab, results from the sliding window analyses were graphed, highlighting the positions across the PCGs of each significant radical property. Under the substitutions (Substs) tab, the “properties by site” tables showed the specific codon, amino acid, and branch position of each significant radical property.

### Phylogenetic analyses

We employed two methods to confirm tree-like structure in the mitogenomic data. Firstly, we used the four-cluster likelihood mapping (FcLM) method [58] implemented in IQ-TREE [59]. The method calculates the maximum likelihood for the three fully resolved tree topologies possible from all unique sequence quartets in the data (using option -lmap ALL). The three corners of an equilateral triangle represent the three fully resolved topologies, and a point within this triangle represents the maximum likelihood value of each topology. Datasets with strong phylogenetic content have most points mapped to fully resolved regions of the triangle (corners), while those with a low phylogenetic signal have a significant proportion of points mapped to unresolved regions of the triangle (center). Secondly, we used the NeighborNet algorithm implemented in SplitsTree [60] to construct a phylogenetic network and assess whether the data nature was tree-like, with a bifurcating phylogeny, or network-like, with reticulation and conflicting phylogenetic signals.

Maximum likelihood phylogenetic analyses were performed on PCG and PCG + rRNA alignments. Maximum likelihood gene trees were inferred using IQ-TREE [59] and optimal models (-mset mrbayes) found using ModelFinder [61]. Selection of an appropriate outgroup followed the recommendations of Grant [62], by including the closest known sister taxa and successively expanding the outgroup sample until ingroup topology was shown to be stable in at least two iterations. Outgroup sampling included specimens from the subgenera *Nyssorhynchus* (*An. braziliensis*, NC037791; *An. darlingi*, NC014275; *An. evansae*, MF381711; *An. nuneztovari*, MF381680; *An. strodei*, NC037808), *Anopheles* (*An. minor*, MF381684), *Kerteszia* (*An. cruzii*, NC024740; *An. homunculus*; NC030248), and *Stethomyia* (*An. kompi*, NC037827; *An. nimbus*, NC037811). The TIGER [Tree Independent Generation of Evolutionary Rates] method of Cummins and McInerney [63] was used to identify the most rapidly evolving sites in the PCG alignment, which may be a source of noise with little phylogenetic signal. Sites were placed into 10 bins, and those in the fastest bin (bin 10) were removed. The resulting “slow site” alignment was then input for phylogenetic analysis, as above.

### Species tree and species delimitation

StarBEAST2 [64] was used to construct a time-calibrated species tree using the multispecies coalescent method. Divergence times among species in the complex were estimated using the insect mitochondrial substitution rate of 0.0115 mutations/nucleotide/million years described by Brower [65]. All PCGs were considered linked across tree, site, and clock models. A strict and an uncorrelated log-normal relaxed clock model was run, using a Yule tree prior, analytical population size integration, and an uninformative prior (1/*x*) for population size. The site model was set to the optimal model for ingroup sequences: HKY+I+G. The analysis was run for 1 × 10^8^ steps with sampling every 5 × 10^4^ steps. Adequate mixing was achieved by ensuring that the effective sample size (ESS) of all parameters was greater than 200. Duplicate runs were performed to check for consistency.

The unguided Bayesian species delimitation method (A11 model; [66]), implemented in Bayesian Phylogenetics and Phylogeography (BPP; [67]), was used to jointly estimate the species tree and the species delimitation using the multispecies coalescent model and reversible-jump Markov chain Monte Carlo (rjMCMC) analyses in a Bayesian framework. Population size parameter (*θ*) was assigned an inverse-gamma prior run for different values: IG(3, 0.005), IG(3, 0.01), and IG(3, 0.02), i.e., IG(*α*, *β*), giving a mean of *m* = *β*/(*α* − 1), or mean species nucleotide diversity of 0.25, 0.,5 and 1.0%. We considered these to be a realistic range of estimates for diversity in the Albitarsis Complex, where mean nucleotide diversity among species was estimated at 0.87%, based on *COI* barcodes sequenced in Motoki et al. [11]. Divergence times at the root of the species tree (*τ*) were assigned inverse-gamma priors IG(3, 0.04), IG(3, 0.05), and IG(3, 0.06), giving means for τ of 0.02, 0.025, and 0.03, i.e., 2–3% sequence divergence between the root of the species tree and the present time. This estimate is comparable to *p*-distance derived from a midpoint-rooted neighbor-joining tree (2.1%). Population size (*θ*) and divergence time (*τ*) priors were denoted as low (*θ*_low_, *τ*_low_), medium (*θ*_med_, *τ*_med_), and high (*θ*_high_, *τ*_high_). Duplicate runs were performed using both rjMCMC algorithm 0 (with *∈* = 2) and 1 (with *α* = 2 and *m* = 1) to check for consistency. The importance of the starting tree on output was also tested using the maximum likelihood topology tree and a random topology tree. For each run, 100,000 samples were collected by sampling every five iterations after a burn-in of 20,000 iterations.

## Results

**Fig. 1:**
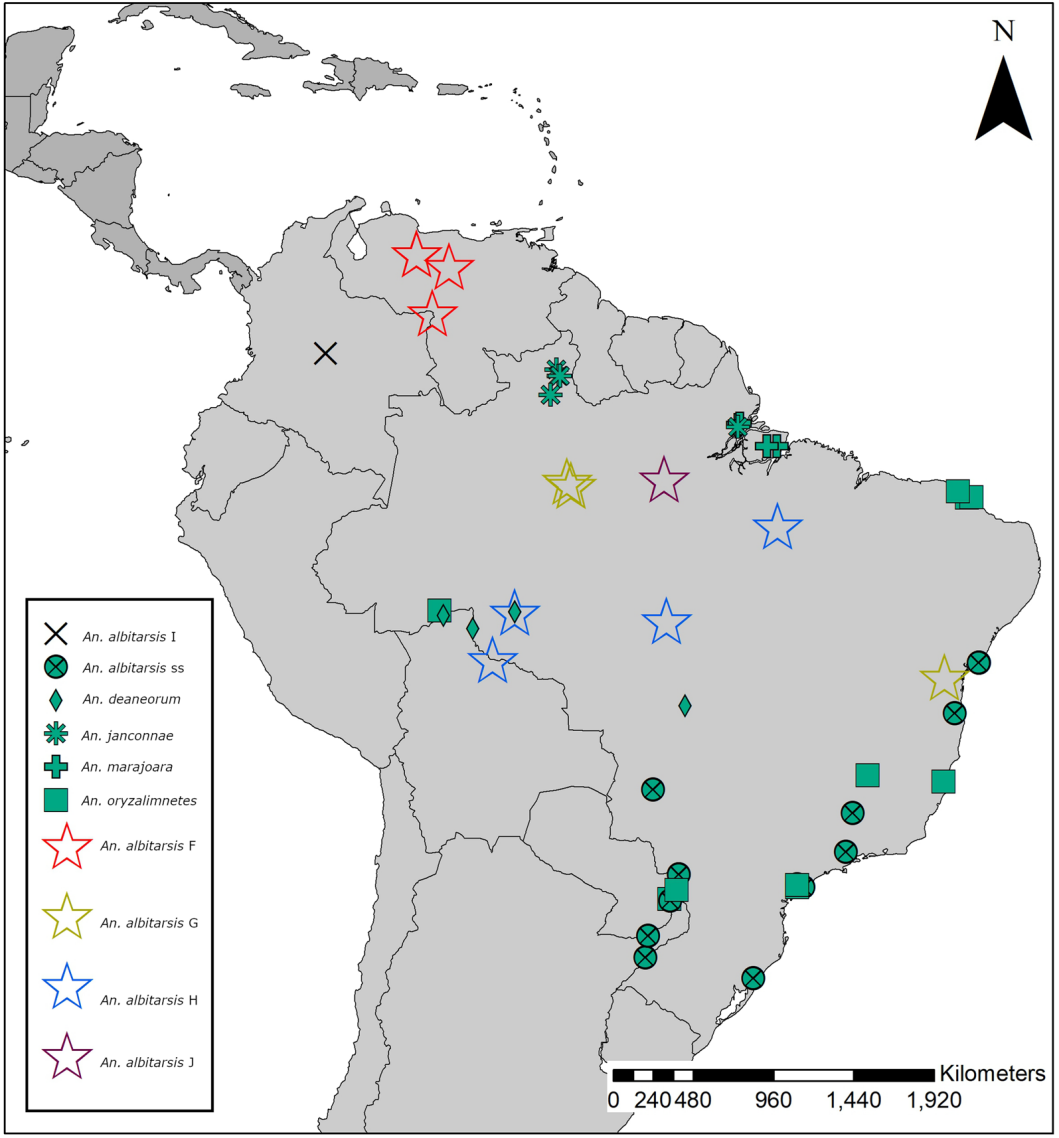
Collection localities for the Albitarsis Complex specimens. For illustrative purposes, locations have been jittered to reduce overplotting

**Fig. 2:**
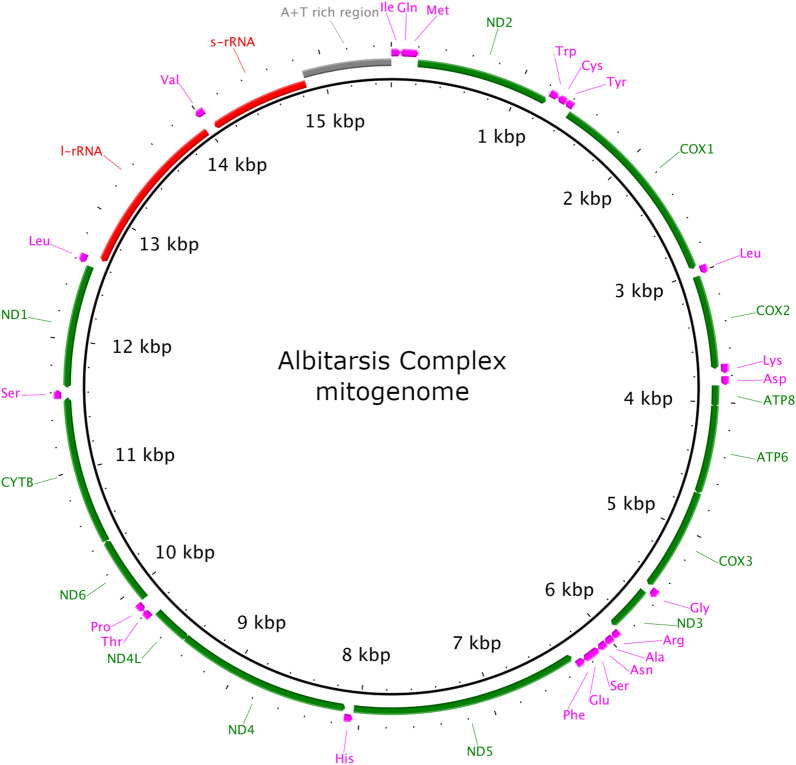
Mitochondrial genome structure of the Albitarsis Complex mitogenome. All 13 protein-coding genes (green), 22 tRNA genes (pink), two rRNA genes (red), and the AT-rich control region (gray) are indicated. Arrows indicate the direction of transcription

The geographical distribution of specimens analyzed in this study can be seen in Fig. 1. The complete mitogenomes from the Albitarsis Complex comprised 13 PCGs, two rRNA genes, 22 tRNA genes, and an AT-rich region (Fig. 2). Mitogenome lengths varied from 15,206 base pairs (bp) in *An. janconnae* 51912405, to 15,525 bp in *An. deaneorum* MF381590 (an exception was *An. janconnae* 51912453, which was 14,047 bp in length and suffered from low coverage and poor assembly around the AT-rich region). Gene composition and order in the mitogenomes were consistent across all species (Fig. 2; Additional file 2: Table S2).

### Selection

**Fig. 3:**
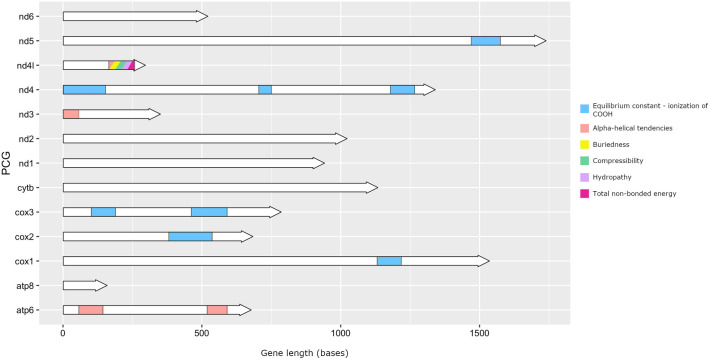
TreeSAAP results showing regions among the 13 protein-coding genes under positive selection (*P*-value < 0.001). Gene lengths are taken from sequence alignments

TreeSAAP sliding window analysis indicated six physicochemical properties undergoing positive selection in eight of the 13 PCGs (Fig. 3). Positive selection in the equilibrium constant (ionization of COOH) property was found to be the most widespread, occurring in five of the PCGs. Positive selection in alpha-helical tendencies was detected in *ATP6* and *ND3*, while positive selection for the remaining four physicochemical properties occurred in a short 94-bp section of the *ND4l* gene. When mapping physicochemical property changes to branch position, positive selection was detected at four internal branches (considered true candidate variants of positive selection). One of these, a change from serine to leucine at the *ND2* gene and increasing chromatographic index, was unique to *An. janconnae*. Another, a change from isoleucine to asparagine at the *ND5* gene and increasing polarity, was unique to *An. albitarsis* G. The remaining two changes, serine to phenylalanine at the *ND1* gene (increasing chromatographic index and solvent-accessible reduction ratio) and glutamate to lysine at the *ND3* gene (increasing isoelectric point), were associated with intraspecific branches in the *An. albitarsis* and *An. oryzalimnetes* clades, respectively.

Prior to undertaking phylogenetic analyses, we assessed alignments for variability and checked codon position 3 among PCGs for substitution saturation (Additional file 3: Table S3; Additional file 4: Table S4). The PCG alignment had considerably more information content (13 genes; parsimony-informative sites = 10–195) than the rRNA and tRNA alignments. The tRNA alignment was least informative (22 genes; parsimony-informative sites = 0–3), and was excluded from subsequent phylogenetic analyses. No signal of substitution saturation at codon position 3 was detected in the PCG alignment.

### Network- versus tree-likeness

**Fig. 4:**
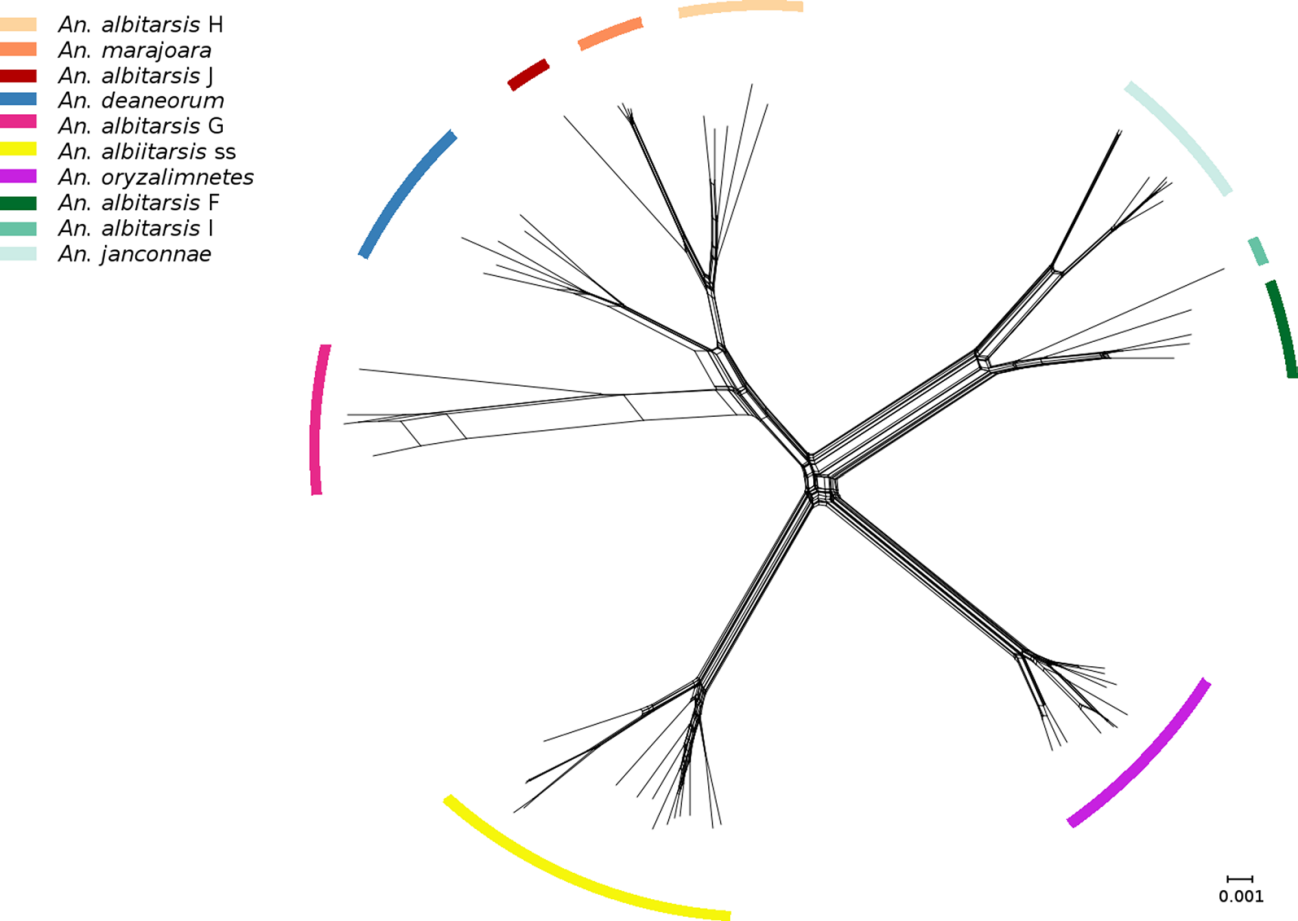
SplitsTree (NeighborNet) network analysis of the 13 protein-coding genes of the Albitarsis Complex, showing the tree-like nature of the mitogenome data

The construction of a NeighborNet graph using both PCG and PCG + rRNA alignments showed a clear tree-like structure to the data (Fig. 4; Additional file 5: Figure S1). Similarly, FcLM analysis for the two sets of alignments found strong tree-likeness in the data, with more that 96% of all topologies found in tree-like regions of the map (Additional file 6: Figure S2; Additional file 7: Figure S3).

### Mitogenome gene tree

**Fig. 5:**
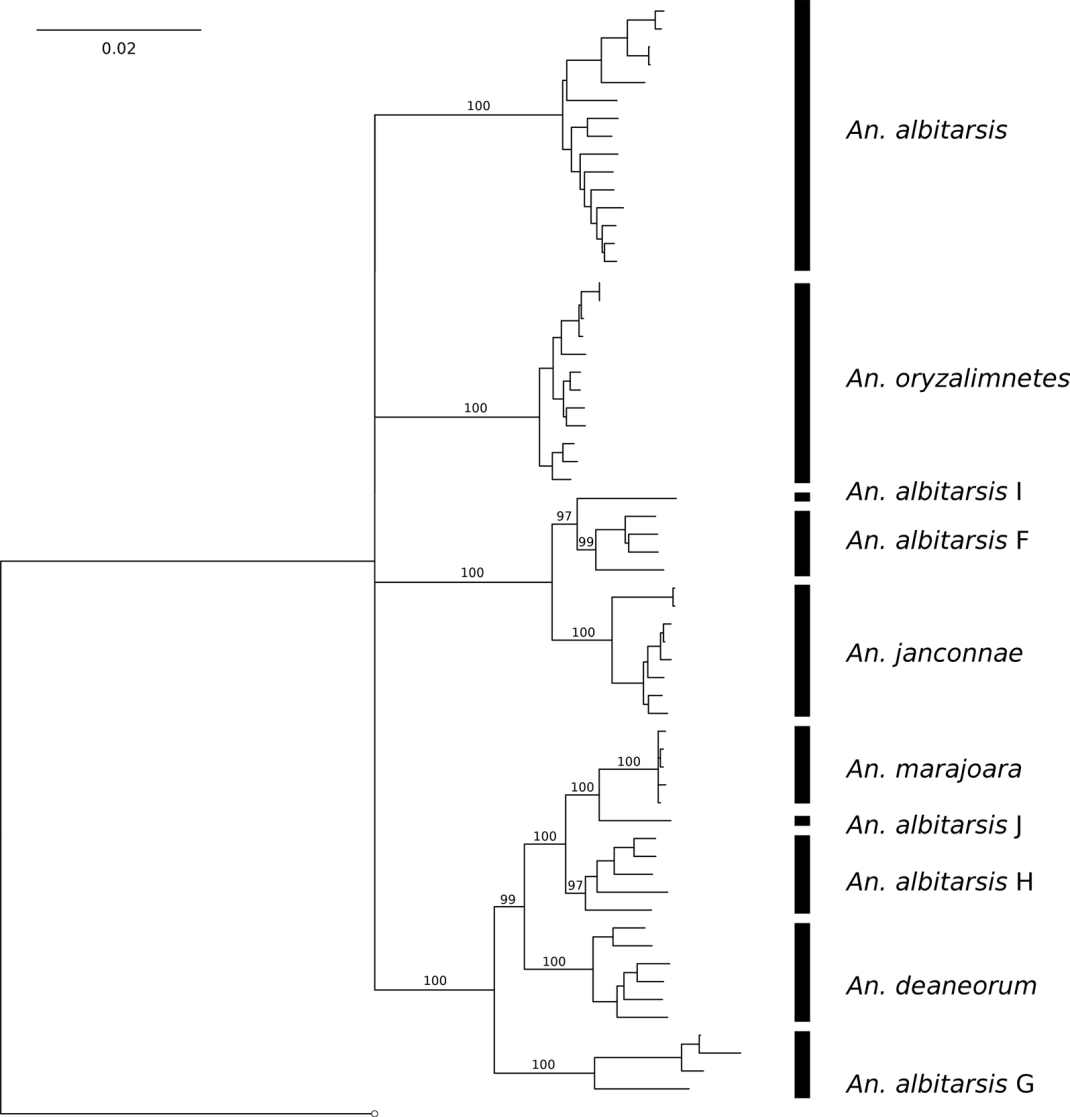
Maximum likelihood (70% majority-rule bootstrap consensus) gene tree of 13 PCGs from the Albitarsis Complex mitogenome. Bootstrap support is shown for each of the 10 members of the complex

Iterative expansion of outgroup samples did not substantially alter ingroup topology and support. The first iteration of the outgroup (*An. braziliensis*, *An. darlingi*, and *An. strodei*) was therefore the one used in subsequent phylogenetic analyses. Maximum likelihood trees constructed from both PCG and PCG + rRNA alignments yielded a consistent tree topology (Fig. 5 and Additional file 8: Figure S4, respectively). The 10 species of the Albitarsis Complex mitogenomes were resolved. Eight of these 10 species formed strongly supported (≥ 97% bootstrap support, BS) monophyletic clades. The remaining two species, *An. albitarsis* I and *An. albitarsis* J, were each represented by a single mitogenome, and so the monophyly of these two species could not be properly tested. The most basal split in the tree formed a four-way polytomy. The clades formed from this polytomy comprised *An. albitarsis* (100% BS), *An. oryzalimnetes* (100% BS), *An. albitarsis* F + *An. albitarsis* I + *An. janconnae* (100% BS), and *An. albitarsis* G + *An. albitarsis* H + *An. albitarsis* J + *An. deaneorum* + *An. marajoara* (100% BS). PCG and PCG + rRNA alignments with exclusion of the fastest-evolving sites yielded the same topology as the whole datasets, with comparable levels of support across the trees (Additional file 9: Figure S5).

### *COI* and *COI* barcode gene trees

**Fig. 6:**
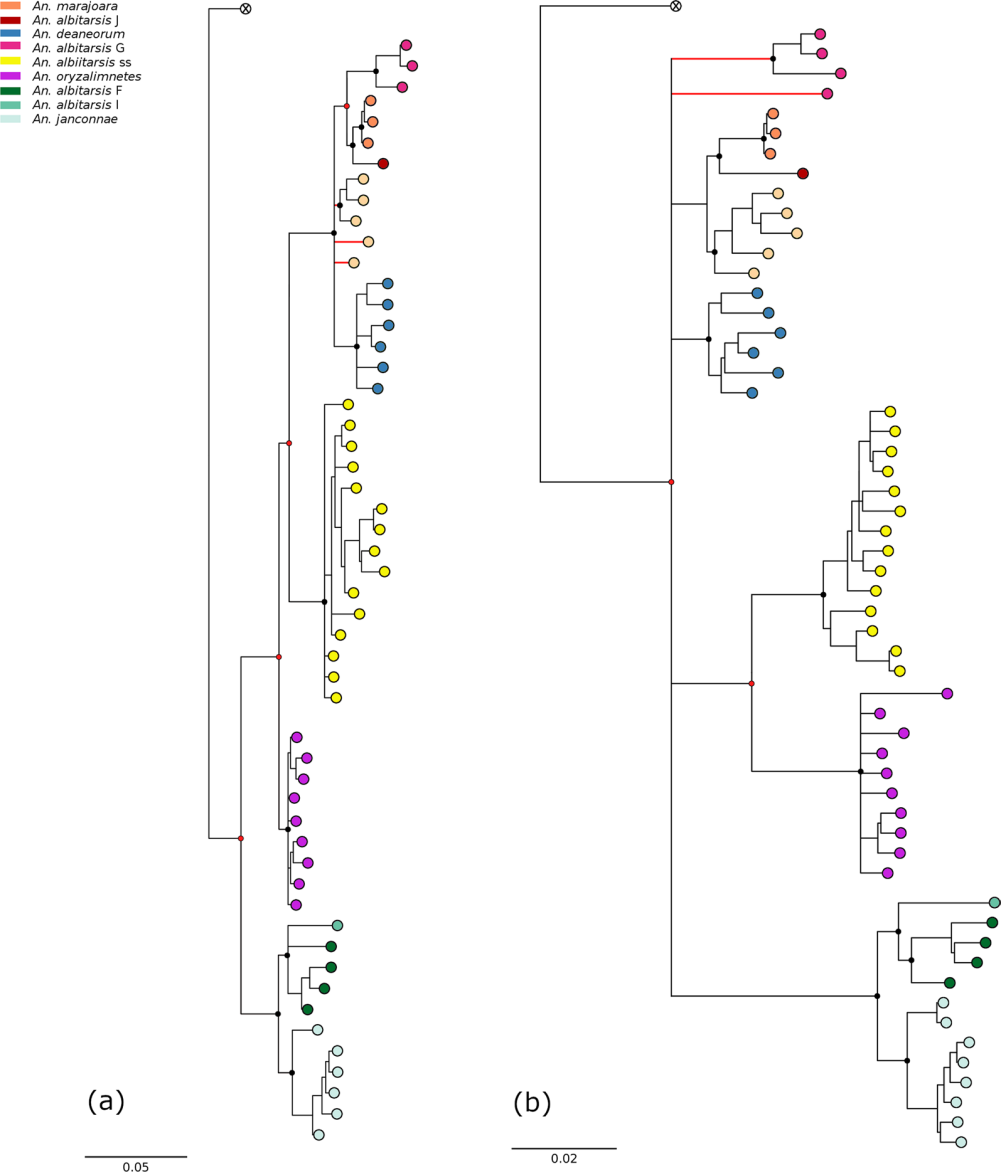
Maximum likelihood (70% majority-rule bootstrap consensus) gene trees for (**a**) *COI* barcode and (**b**) *COI* data. Discordance with the mitogenome gene tree highlighted as red nodes and branches

Maximum likelihood trees of both the *COI* gene and the *COI* barcode region yielded considerably different topologies from that of the mitogenome. Discordance between *COI* trees and the mitogenome tree are represented by red nodes, where splits and polytomies differ, and red branches, where species clades are not monophyletic (Fig. 6a, b). In the *COI* tree (Fig. 6b), *An. albitarsis* and *An. oryzalimnetes* formed a new sister relationship in a separate clade, while the *An. albitarsis* G + *An. albitarsis* H + *An. albitarsis* J + *An. deaneorum* + *An. marajoara* clade found in the mitogenome phylogeny fragmented and formed part of a six-way basal polytomy. The *An. albitarsis* H + *An. albitarsis* J + *An. marajoara* clade was retained, while *An. albitarsis* G was found to be no longer monophyletic. The *COI* barcode tree (Fig. 6a) displayed greater discordance with the mitogenome phylogeny than the *COI* tree. The *An. albitarsis* F + *An. albitarsis* I + *An. janconnae* clade, which was found in the basal four-way polytomy in the mitogenome tree, was here found as a sister to a clade formed by all remaining species in the *COI* barcode tree. Within this sister clade, *An. oryzalimnetes* is sister to a clade containing the remaining species in this clade, and within this latter clade, *An. albitarsis* sensu stricto is sister to a multifurcating clade containing *An. albitarsis* G + *An. albitarsis* H + *An. albitarsis* J + *An. deaneorum* + *An. marajoara*. Within this clade, *An. albitarsis* H, which formed a strongly supported clade in the mitogenome tree, fragmented into the polytomy.

### Species tree and species delimitation

The joint species tree and species delimitation BPP analysis found a high degree of support for species designations in the Albitarsis Complex, but there was little information in the data to infer species phylogeny under a multispecies coalescent model. The differences between output produced from Algorithm 0 and Algorithm 1 and from varying τ were also negligible (Table 1), and results described refer to those produced from Algorithm 0 and *τ*_med_. The posterior probability (PP) for all 10 species ranged from 0.88 to 0.95 (given range refers to *θ*_high_–*θ*_low_) and for nine species ranged from 0.05 to 0.12. There were three alternative nine-species partitions, which merged *An. albitarsis* F with *An. albitarsis* I (PP = 0.02–0.04), *An. albitarsis* J with *An. albitarsis* H (PP = 0.02–0.03), or *An. albitarsis* J with *An. marajoara* (PP = 0.01–0.05). Although the 10-species partition was therefore the most strongly supported, it did not exceed the 0.95 PP threshold considered strong support for species splitting [68]. In addition, the uncertainty in the 10-species partition was due to the placement of *An. albitarsis* I and *An. albitarsis* J, both of which are represented by a single specimen.

**Table 1:** BPP analysis that jointly estimates the species tree and species delimitation in the Albitarsis Complex

*Algorithm 0*								
*τ* ~ IG(3, 0.04)								
*θ*	10-species	9-species (total)	9-species (albF-albI)	9-species (albJ-albH)	9-species (albJ-mara)	Best tree posterior probability	MAP tree	No. trees
IG(3,0.02)	0.87	0.12	0.04	0.03	0.05	0.03 (10-species)	((((alb_F, alb_I), jan), (alb_G, ((alb_H, (alb_J, mara)), dean))), (alb, ory));	4746
IG(3,0.01)	0.92	0.08	0.04	0.02	0.02	0.11 (10-species)	((((alb_F, alb_I), jan), (alb, ory)), (alb_G, ((alb_H, (alb_J, mara)), dean)));	658
G(3,0.005)	0.95	0.05	0.03	0.01	0.01	0.33 (10-species)	((((alb_F, alb_I), jan), (alb, ory)), (alb_G, ((alb_H, (alb_J, mara)), dean)));	73

*τ* ~ IG(3, 0.05)								
*θ*	10-species	9-species (total)	9-species (albF-albI)	9-species (albJ-albH)	9-species (albJ-mara)	Best tree posterior probability		No. trees
IG(3,0.02)	0.88	0.12	0.04	0.03	0.05	0.03	((((alb_F, alb_I), jan), (alb, ory)), (alb_G, ((alb_H, (alb_J, mara)), dean)));	4731
IG(3,0.01)	0.92	0.08	0.03	0.03	0.02	0.11	((((alb_F, alb_I), jan), (alb, ory)), (alb_G, ((alb_H, (alb_J, mara)), dean)));	550
IG(3,0.005)	0.95	0.05	0.02	0.02	0.01	0.30	((((alb_F, alb_I), jan), (alb, ory)), (alb_G, ((alb_H, (alb_J, mara)), dean)));	78

*τ* ~ IG(3, 0.06)								
*θ*	10-species	9-species (total)	9-species (albF-albI)	9-species (albJ-albH)	9-species (albJ-mara)	Best tree posterior probability		No. trees
IG(3,0.02)	0.88	0.12	0.05	0.03	0.04	0.03	((((alb_F, alb_I), jan), (alb_G, ((alb_H, (alb_J, mara)), dean))), (alb, ory));	4894
IG(3,0.01)	0.92	0.08	0.03	0.03	0.02	0.10	((((alb_F, alb_I), jan), (alb, ory)), (alb_G, ((alb_H, (alb_J, mara)), dean)));	693
IG(3,0.005)	0.95	0.05	0.02	0.02	0.01	0.32	((((alb_F, alb_I), jan), (alb, ory)), (alb_G, ((alb_H, (alb_J, mara)), dean)));	171

*Algorithm 1*								
*τ* ~ IG(3, 0.04)								
*θ*	10-species	9-species (total)	9-species (albF-albI)	9-species (albJ-albH)	9-species (albJ-mara)	Best tree posterior probability		
IG(3,0.02)	0.88	0.12	0.04	0.02	0.04	0.03	((((alb_F, alb_I), jan), (alb_G, ((alb_H, (alb_J, mara)), dean))), (alb, ory));	5066
IG(3,0.01)	0.92	0.08	0.04	0.02	0.02	0.11	((((alb_F, alb_I), jan), (alb, ory)), (alb_G, ((alb_H, (alb_J, mara)), dean)));	658
IG(3,0.005)	0.95	0.05	0.02	0.02	0.01	0.31	((((alb_F, alb_I), jan), (alb, ory)), (alb_G, ((alb_H, (alb_J, mara)), dean)));	79

*τ* ~ IG(3, 0.05)								
*θ*	10-species	9-species (total)	9-species (albF-albI)	9-species (albJ-albH)	9-species (albJ-mara)	Best tree posterior probability		No. trees
IG(3,0.02)	0.88	0.12	0.04	0.02	0.05	0.03	((((alb_F, alb_I), jan), (alb, ory)), (alb_G, ((alb_H, (alb_J, mara)), dean)));	5151
IG(3,0.01)	0.93	0.07	0.03	0.02	0.02	0.11	((((alb_F, alb_I), jan), (alb, ory)), (alb_G, ((alb_H, (alb_J, mara)), dean)));	738
IG(3,0.005)	0.95	0.05	0.02	0.01	0.01	0.32	((((alb_F, alb_I), jan), (alb, ory)), (alb_G, ((alb_H, (alb_J, mara)), dean)));	70

*τ* ~ IG(3, 0.06)								
*θ*	10-species	9-species (total)	9-species (albF-albI)	9-species (albJ-albH)	9-species (albJ-mara)	Best tree posterior probability		No. trees
IG(3,0.02)	0.88	0.12	0.04	0.02	0.04	0.03	((((alb_F, alb_I), jan), (alb, ory)), (alb_G, ((alb_H, (alb_J, mara)), dean)));	4792
IG(3,0.01)	0.92	0.08	0.03	0.02	0.02	0.11	((((alb_F, alb_I), jan), (alb, ory)), (alb_G, ((alb_H, (alb_J, mara)), dean)));	686
IG(3,0.005)	0.95	0.05	0.02	0.01	0.01	0.31	((((alb_F, alb_I), jan), (alb, ory)), (alb_G, ((alb_H, (alb_J, mara)), dean)));	75

*τ* = mean root age, specified by an inverse-gamma prior IG(*a*, *b*). Mean = *b*/(*a* − 1)

*θ* = mean genetic diversity, specified by an inverse-gamma prior IG(*a*, *b*). Mean = *b*/(*a* − 1)

Best tree = maximum a posteriori (MAP) species tree

No. trees = number of trees in the 95% credibility set

Species tree estimation provided a 95% credibility set of between 70 and 5151 unique species tree topologies (Table 1). The maximum a posteriori (MAP) species tree had a PP of just 0.03–0.33. The species tree is, therefore, highly uncertain under a model of multispecies coalescence, and the mitogenome appears to lack sufficient information to fully resolve the species phylogeny.

A strict- and relaxed-clock species tree were construction in StarBEAST2 (Additional file 10: Figure S6; Additional file 11: Figure S7). For the relaxed-clock species tree, clock rate variation among branches was low (posterior branchRatesStdev.Species mean < 0.2) and 95% highest posterior density (HPD) intervals for relative clock rates in all branches included 1, indicating the strict-clock species tree was more appropriate for this dataset. However, both clock models produced the same significantly supported (> 70% PP) clades, and similar divergence time estimates (< 5% differences). The root of the Albitarsis Complex was dated to approximately 2.63 million years ago (mya: 95% HPD: 1.90–3.19 mya; Table 2). The *An. albitarsis* F + *An. albitarsis* I + *An. janconnae* clade is the only clade within the Albitarsis Complex found to reach moderate levels of support in the species tree (79% PP). Divergence within this clade is dated to approximately 1.01 mya (95% HPD: 0.64–1.35 mya; Table 2). All remaining splits in the species tree were insufficiently supported (< 70% PP).

**Table 2:** Estimated divergence times in the Albitarsis Complex from StarBEAST2 analysis

Clade	Age, mean (mya)	Age, 95% HPD (mya)	Posterior support (%)
Albitarsis Complex	2.63	1.90–3.19	100
*An. janconnae* + *An. albitarsis* F + *An. albitarsis* I	1.01	0.64–1.35	79

## Discussion

Describing species diversity and relationships in the Albitarsis Complex is of great importance to malaria vector control initiatives in South America. Delivering targeted control measures, often with limited resources, relies on an ability to identify and incriminate vectors and discriminate them from benign relatives, and being cognizant of various biological processes, such as adaptive introgression, in vector complexes [69]. Additionally, understanding how diversity has been shaped by historical and ecological factors is not merely an academic exercise—it can provide important insights into how species of public health importance will respond to future events, such as deforestation, climate change, and rapid human population expansion. Here, we present a description of mitogenomic diversity in the Albitarsis Complex, and describe phylogenetic relationships among its species.

The gene composition and order of mitogenomes was conserved among species of the Albitarsis Complex, and was consistent with the architecture of mitogenomes from other species in the anopheline subgenera *Nyssorhynchus* (*An. aquasalis*) [37], *Cellia* (*An. stephensi* and *An. dirus*) [70], *Kerteszia* (*An. bellator*, *An. cruzii*, *An. homunculus*, and *An. laneanus*) [71] and *Anopheles* (*An. sinensis*) [72].

We found only signatures of positive selection among genes in the mitogenome. The most widespread of these were changes to the equilibrium constant in two ND genes and all COX genes, which are associated with complexes I and IV of the oxidative phosphorylation (OXPHOS) system, respectively, and its various cellular functions and metabolic processes. However, in the current study we considered only property changes that could be mapped to internal branches of the mitogenome tree to be true candidate variants under positive selection, as property changes at terminal tips may represent deleterious changes prior to the action of purifying selection. Increased chromatographic index and polarity properties were mapped to the *An. janconnae* and *An. albitarsis* G clades, respectively. These properties have the potential to influence residue water solubility and reactive oxygen species (ROS) production, which can influence an organism’s longevity and immune response [73–75] and defense against viral and microbial pathogens [76, 77]. Such positive selection may have played an important role in the rapid diversification and adaptation in the complex, which occurred across much of the southern Americas over a comparatively short period of time. It may also have been an important adaptive response to infection by pathogens such as *Plasmodium*, which is believed to reduce mosquito survival [78].

The mitogenome gene tree constructed in this study supported the resolution of all 10 members of the Albitarsis Complex, a finding that is largely consistent with the *COI* barcode clustering of Motoki et al. [11] and Ruiz et al. [7]. Although mitogenomic genes are expected to share the same evolutionary tree, we may expect to see topological variation among gene trees due to systematic error (as opposed to biological process), and we observe this in the gene trees from this study and among other studies [7, 11]. An important feature of the mitogenome gene tree is the basal polytomy, which contains four main clades. This is likely a soft polytomy, attributable to the considerable diversification that occurred over a short period of time and the insufficient information in the data to resolve the branching order. All clades derived from this four-way polytomy are very highly supported. Considerable differences are found between this tree and the *COI* and *COI* barcode gene trees, where some putative species (*An. albitarsis* G and *An. albitarsis* H) fragment into polytomies, and topological conflict is found at higher levels in the trees. Differences can also be seen in comparisons to the findings of other studies. The Bayesian *COI* tree of Ruiz et al. [7] displays several differences when only strongly supported branches are considered (i.e., a four-way polytomy in the *An. albitarsis* G + *An. albitarsis* H + *An. deaneorum* + *An. marajoara* clade), while the *COI* tree of Motoki et al. [11] recovers *An. albitarsis* H as a strongly supported sister taxon to *An. deaneorum*, in contrast to the mitogenome gene tree, which finds it to be a sister of the *An. marajoara* + *An. albitarsis* J clade. Given these findings, although *COI* and *COI* barcode data are routinely used for species delimitation analyses, it appears that these data do not effectively recovery the topology of the Albitarsis Complex mitogenome gene tree.

A highly important consideration when inferring the evolutionary relationships of recently diverged species is to account for the retention of ancestral polymorphism or incomplete lineage sorting, which creates discordance between gene trees and species trees. This discordance becomes more pronounced when divergence times are short relative to the effective population sizes [79], as is likely the case in our study. Our main phylogenetic inferences for species in the complex therefore relied on the multispecies coalescent model, which provides more accurate assessment of uncertainty in the species tree estimate and better estimates of species divergence times, while accounting for incomplete lineage sorting.

Due to the comparatively recent evolutionary history of the Albitarsis Complex, we were unable to use appropriate geologically dated fossils or phylogeographical events to establish an absolute timescale for divergence. The fossil record for mosquitoes is particularly scant. Only two fossil anopheline mosquitoes, *An.* (*Nyssorhynchus*?) *dominicanus* and *An*. (?) *rottensi*, currently exist and date from approximately 15–45 mya and 25 mya, respectively [80, 81]. We therefore used the mitochondrial DNA clock of Brower [65], derived from South American *Heliconius* butterflies. We considered Brower’s [65] estimate to be suited to our study as it was based on calibration points since the late Pliocene (i.e., comparable to our study’s timescale), and on a range of fast and slow genes from across the mitogenome. Papadopoulou et al. [82] found that their mitochondrial substitution rate for Coleoptera, which used older calibration points, was also comparable to that of Brower [65]. However, we recognize and here underline the importance of undertaking further work which seeks to test the utility of this clock at lower levels in *Anopheles* using temporally appropriate calibration points from geological, biogeographical, and/or paleoclimatic data.

Unfortunately, there was insufficient information in the mitogenome to provide strongly supported divergence estimates among most species in the Albitarsis Complex. While the inability to resolve basal relationships in the species tree was also found in the mitogenome gene tree, the former also displays a considerable amount of uncertainty at lower levels of the species tree. Despite these shortcomings, the initial radiation of the Albitarsis Complex could be dated to approximately 2.63 mya, or around the Pliocene/Pleistocene boundary (2.58 mya; [83]). It appears plausible that the comparatively recent origin of this diverse (putatively 10 species) complex required some degree of adaptation to the considerable climatic and environmental changes occurring from the late Pliocene [84] through to the late Pleistocene [85] on the continent. Our detection of positive selection in some species appears to support this. Our estimates for divergence in the Albitarsis Complex were not explained by marine incursions, which occurred no later than the end of the Miocene, approximately 5.33 mya [86]. It has been suggested that the Amazon River may have played an important role in driving the initial radiation in the complex [19]. According to our findings, the Amazon River barrier hypothesis also does not in itself explain this radiation, as the formation of the Amazon River, approximately 5–12 mya, predates initial diversification in the complex by at least approximately 2 million years. It is possible that the Amazon River acted as a subsequent barrier to the range expansion of locally adapted populations from either side of the river. However, the strength of these claims relies on the ability of the Amazon River to act as a barrier to dispersal and gene flow. The fact that all species inhabiting the banks of the Amazon, with the exception of *An. albitarsis* J, have now been collected on both sides of the river (shown for *An. janconnae* and *An. marajoara* in Motoki et al. [11], and for *An. albitarsis* G and *An. oryzalimnetes* in McKeon et al. [39]) would appear to refute these hypotheses, although human-aided transport across the Amazon River cannot yet be excluded as a possible explanation for these occurrences.

Our findings suggest that divergence in the *An. albitarsis* F + *An. albitarsis* I + *An. janconnae* clade began between 0.64 and 1.35 mya. This period was also highly important in shaping interspecific diversification in other mosquito complexes (Nuneztovari Complex) [87] and forest birds [88] and intraspecific clade diversification in forest species of amphibians [89], birds [90], and mammals [91] in northern South America. One of the species that emerged, *An. janconnae*, is an important malaria vector [18] and appears to have undergone considerable adaptation during this period, displaying signatures of positive selection in the mitogenome and comparatively strong habitat specialization, seeking larval habitats with low sun exposure and high water velocities [92].

Although our study does not reject the 10-member Albitarsis Complex hypothesis, it should be noted that the multispecies coalescent approach, along with delimiting species boundaries, may also detect population splits [93]. So, while our delimitation approach provides further support for those formally recognized species in the complex [27], the delimitation of the four putative species (*An. albitarsis* G, H, I, and J) for which no extra-mitogenomic information exists cannot be considered as clear support for their species status. To date, four of the 10 members of the Albitarsis Complex have been defined on the basis of *COI* data only. *Anopheles albitarsis* G (*An. marajoara* lineage 2 in McKeon et al. [39]), *Anopheles albitarsis* J (found as the C3+C4+C5 clade in Lehr et al. [32] and included in McKeon et al. [39]), and *An. albitarsis* I (denoted “near *An. janconnae*” in Gutiérrez et al. [36]) are resolved at the mitochondrial *COI* gene, but not at the nuclear white or *ITS2* genes [7, 11, 36, 39], while *An. albitarsis* H has only ever been analyzed and resolved at the *COI* gene [7]. Examination of GenBank *COI* data shows that minimum interspecific pairwise distances for these four putative species are extremely low (*An. albitarsis* H KJ492691 and *An. albitarsis* J DQ076220 = 1.21% K2P distance; *An. albitarsis* J JQ615466 and *An. marajoara* JQ615442 = 1.55%; *An. albitarsis* I JQ615193 and *An. albitarsis* F JQ615020 = 2.02%), and many do not exceed their maximum intraspecific pairwise distance (*An. albitarsis* H JQ615154 and KJ492402 = 2.00%; *An. albitarsis* F JQ615005 and JQ615009 = 2.66%). Additionally, in our study, *An. albitarsis* I and *An. albitarsis* J are each represented by only a single mitogenome, which is likely to have affected population size estimates in the multispecies coalescent construction of the species tree and delimitation. These species also have the lowest levels of delimitation support, primarily due to the albeit poor support for merging of *An. albitarsis* I with *An. albitarsis* F and *An. albitarsis* J with *An. albitarsis* H or *An. marajoara*. Given the lack of external (extra-mitogenomic) support to form a strong a priori species hypothesis for *An. albitarsis* G, *An. albitarsis* H, *An. albitarsis* I, and *An. albitarsis* J, and the scant sampling of the latter two in the current study, these putative species require particular attention in future studies. These should employ genomic data to discriminate potential population splits from species divergence and be supported by an analysis of morphological and/or ecological datasets before any firm conclusions can be drawn on species designation.

While our study provides important insights into mitogenome architecture, signatures of selection, and the origin and nature of diversification, it also found a considerable degree of uncertainty around the phylogeny of the Albitarsis Complex. A more complete understanding of the origins and diversification of the complex will undoubtedly require genome-wide data, which may help resolve the basal uncertainty, estimate the lower-level branching order, and identify those genomic regions under selection driving incipient speciation. Explaining the origins and nature of species diversity in this complex will also require a much better understanding of the geographical distribution and putative boundaries among its species. Further sampling of its members from prospective habitats, particularly for those flanking the Amazon River, is clearly desirable for more robust testing of biogeographical hypotheses. A comparative study of species radiation in the *Nyssorhynchus* mosquito complexes found across northern South America—which include the Albitarsis Complex, Nuneztovari Complex [94], Oswaldoi-Konderi Complex [95], and those from the Strodei Subgroup [96, 97]—would also be a timely and valuable contribution to understanding the main biogeographical and evolutionary factors driving diversification across many of the most important malaria vectors on the continent.

## Conclusions

These analyses of Albitarsis Complex mitogenomes detected conserved mitogenomic architecture across species as well as signatures of positive selection in *Anopheles janconnae* and *An. albitarsis* G, which may have played a role in the recent and rapid diversification in the Albitarsis Complex. Although considerable uncertainty was found among branching events in trees, the origin of divergence in the Albitarsis Complex was dated to the Pliocene/Pleistocene boundary, approximately 2.63 mya, and divergence within the distinct northern South American clade, comprising *An. albitarsis* F, *An. albitarsis* I, and *An. janconnae,* was estimated at approximately 1 mya. Our findings show that a 10-species hypothesis for the Albitarsis Complex cannot be rejected, but further analysis of the complex using genome-wide data and the inclusion of additional specimens of lesser studied (and as yet still informal) taxa is recommended to further elucidate the origins and diversification of this important assemblage.

## Supplementary Information

**Additional file 1: Table S1.** Geographical origin of the Albitarsis Complex specimens examined in this study.

**Additional file 2: Table S2.** Base composition (% AT content) of the Albitarsis Complex mitogenome, and additional species from the genus.

**Additional file 3: Table S3.** Substitution saturation tests for the third codon position of the 13 PCGs from the Albitarsis Complex mitogenome.

**Additional file 4: Table S4.** Organization, features and alignment statistics of the 13 PCGs, 22 tRNAs and 2 rRNAs from the Albitarsis Complex mitogenome.

**Additional file 5: Figure S1.** SplitsTree (NeighborNet) network analysis of the 13 protein-coding genes and two rRNA genes of the Albitarsis Complex, showing the tree-like nature of the mitogenome.

**Additional file 6: Figure S2.** Four-cluster likelihood mapping of the 13 PCGs of the Albitarsis Complex, showing the tree-like nature of the mitogenome data.

**Additional file 7: Figure S3.** Four-cluster likelihood mapping of the 13 protein-coding genes and two rRNA genes of the Albitarsis Complex, showing the tree-like nature of the mitogenome data.

**Additional file 8: Figure S4.** Maximum likelihood (70% majority-rule bootstrap consensus) gene tree of 13 PCGs and two rRNAs from the Albitarsis Complex mitogenome. Bootstrap support is shown for each of the 10 members of the complex.

**Additional file 9: Figure S5.** Maximum likelihood (70% majority-rule bootstrap consensus) gene tree of (a) PCGs and (b) PCGs and rRNAs from the Albitarsis Complex mitogenome, with the fastest sites removed (Tiger alignments).

**Additional file 10: Figure S6.** Species tree of the 13 PCGs of the Albitarsis Complex under a strict-clock model.

**Additional file 11: Figure S7.** Species tree of the 13 PCGs of the Albitarsis Complex under a relaxed-clock (UCLN) model.

## Data Availability

The Albitarsis Complex mitogenome data that support the findings of this study are available in GenBank, https://www.ncbi.nlm.nih.gov/genbank (MT588297, MW915567, MZ062449–MZ062481).

## Abbreviations

BPP: Bayesian Phylogenetics and Phylogeography
*COI*: Mitochondrial cytochrome c oxidase I
COX: Mitochondrial cytochrome c oxidase
DTT: Dithiothreitol
FcLM: Four-cluster likelihood mapping
MAP: Maximum a posteriori
Mya: Million years ago
ND1–ND6: Nicotinamide adenine dinucleotide dehydrogenase subunit 1–6
PCG: Protein-coding genes
PCR: Polymerase chain reaction
rjMCMC: Reversible-jump Markov chain Monte Carlo
ROS: Reactive oxygen species
rRNA: Ribosomal ribonucleic acid
SDS: Sodium dodecyl sulphate
SEEVA: Spatial evolutionary and ecological vicariance analysis
tRNA: Transfer ribonucleic acid
