# Supplementary material for: Phylogenetic analysis of the Neotropical Albitarsis Complex based on mitogenome data
Source: Parasit Vectors. 2021 Nov 27;14:589. doi: 10.1186/s13071-021-05090-w (PMC8627034; doi:10.1186/s13071-021-05090-w)
Supplement: Supplementary file 5 — Additional file 5: Figure S1. SplitsTree (NeighborNet) network analysis of the 13 protein-coding genes and two rRNA genes of the Albitarsis Complex, showing the tree-like nature of the mitogenome. [file 13071_2021_5090_MOESM5_ESM.pdf]

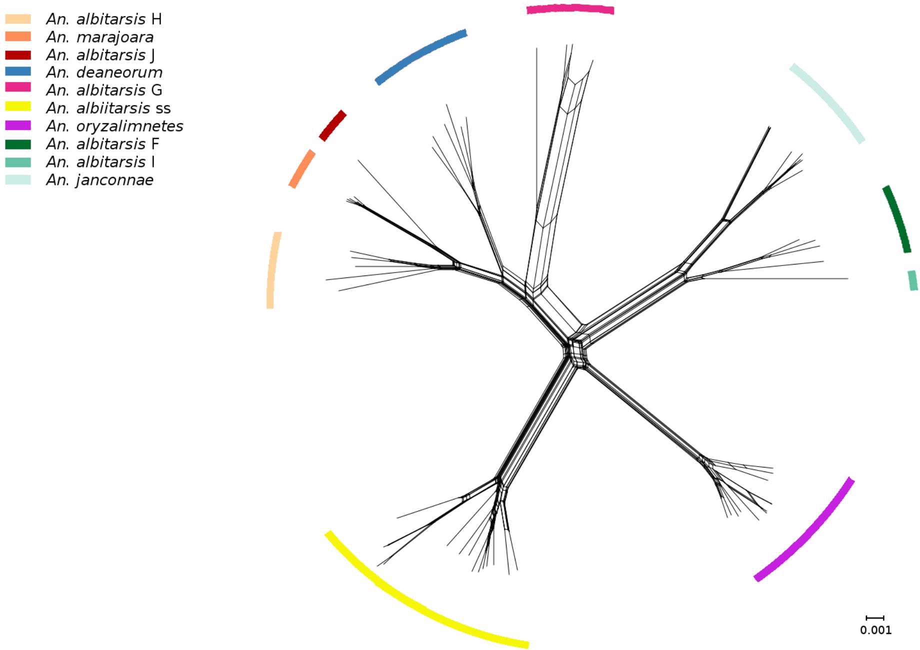
An. albitarsis H
An. marajoara
An. albitarsis J
An. deaneorum
An. albitarsis G
An. albiitarsis ss
An. oryzalimnetes
An. albitarsis F
An. albitarsis I
An. janconnae
0.001
