# Supplementary figures and images for: Phylogenetic analysis of the Neotropical Albitarsis Complex based on mitogenome data
Source: Parasit Vectors. 2021 Nov 27;14:589. doi: 10.1186/s13071-021-05090-w (PMC8627034; doi:10.1186/s13071-021-05090-w)

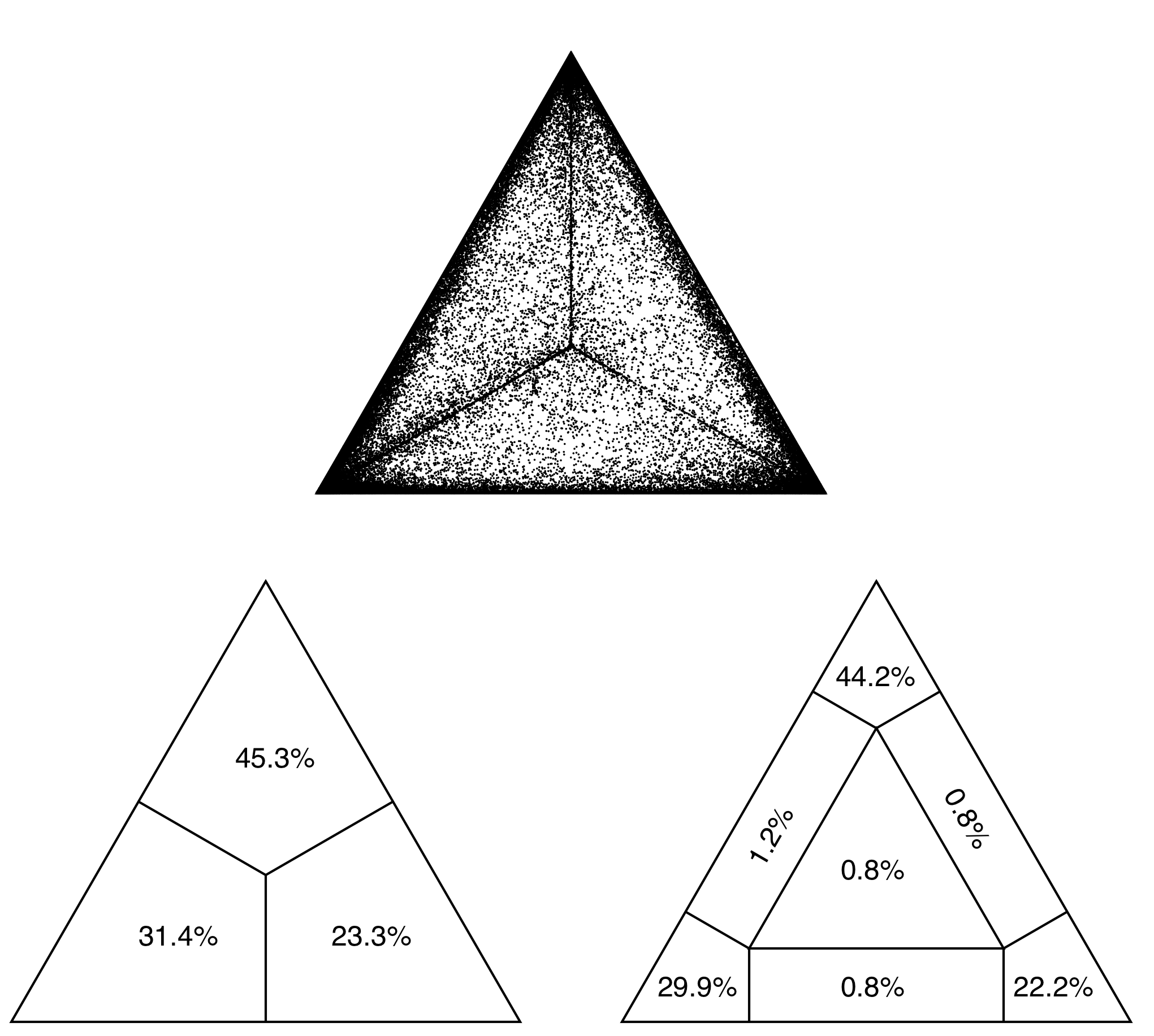

Supplement: Supplementary file 6 — Additional file 6: Figure S2. Four-cluster likelihood mapping of the 13 PCGs of the Albitarsis Complex, showing the tree-like nature of the mitogenome data. [file 13071_2021_5090_MOESM6_ESM.tiff]

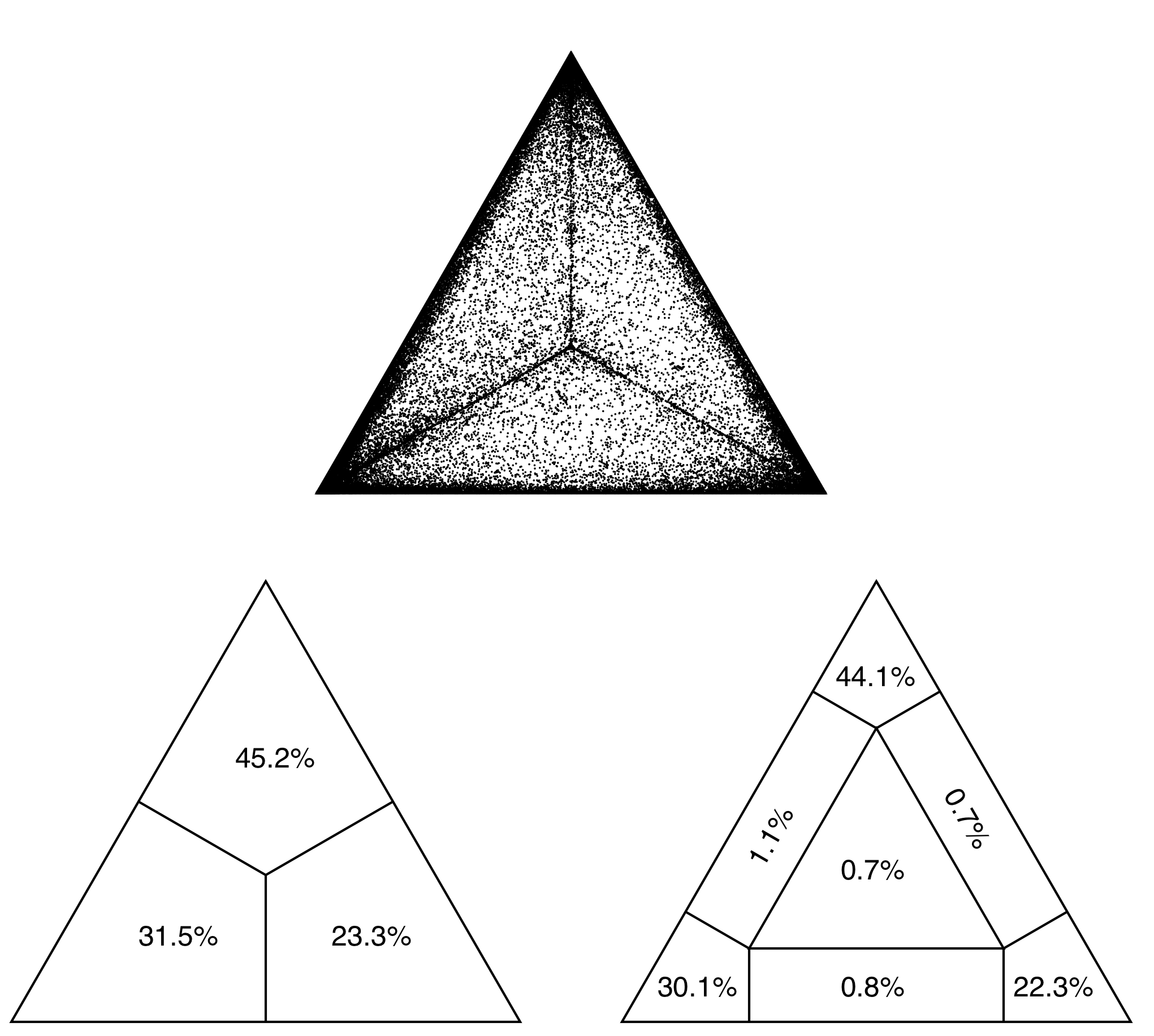

Supplement: Supplementary file 7 — Additional file 7: Figure S3. Four-cluster likelihood mapping of the 13 protein-coding genes and two rRNA genes of the Albitarsis Complex, showing the tree-like nature of the mitogenome data. [file 13071_2021_5090_MOESM7_ESM.tiff]

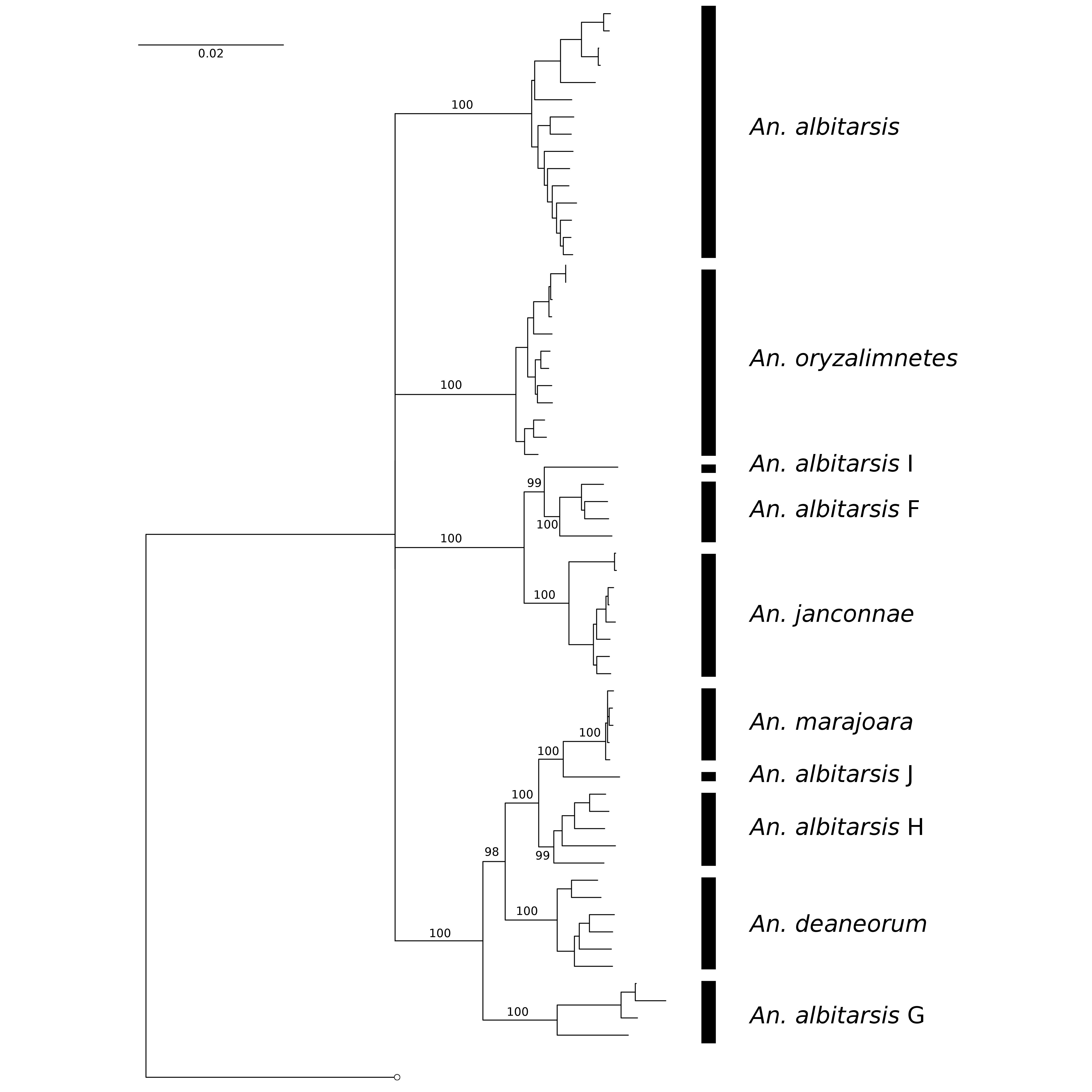

Supplement: Supplementary file 8 — Additional file 8: Figure S4. Maximum likelihood (70% majority-rule bootstrap consensus) gene tree of 13 PCGs and two rRNAs from the Albitarsis Complex mitogenome. Bootstrap support is shown for each of the 10 members of the complex. [file 13071_2021_5090_MOESM8_ESM.png]

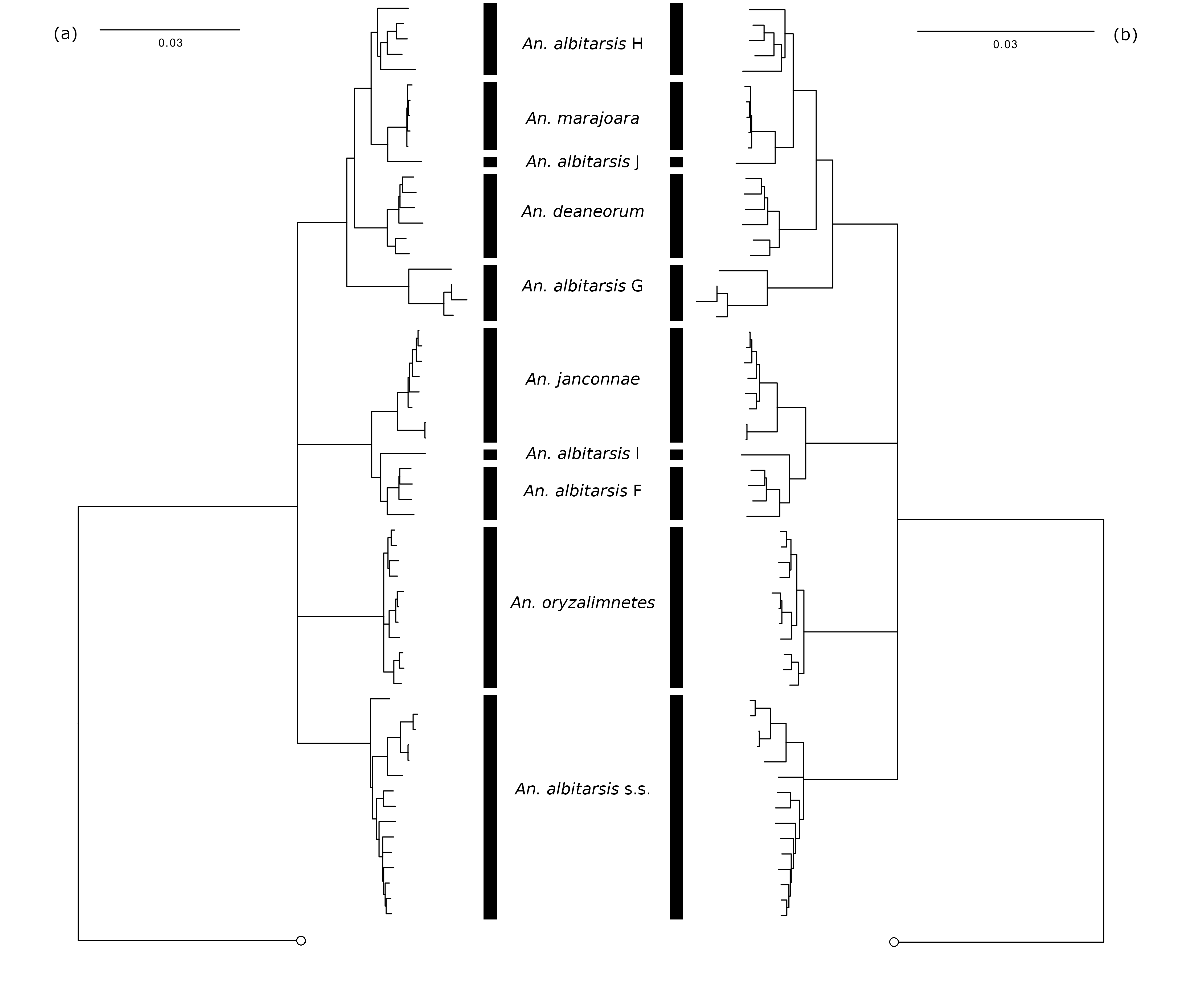

Supplement: Supplementary file 9 — Additional file 9: Figure S5. Maximum likelihood (70% majority-rule bootstrap consensus) gene tree of (a) PCGs and (b) PCGs and rRNAs from the Albitarsis Complex mitogenome, with the fastest sites removed (Tiger alignments). [file 13071_2021_5090_MOESM9_ESM.png]

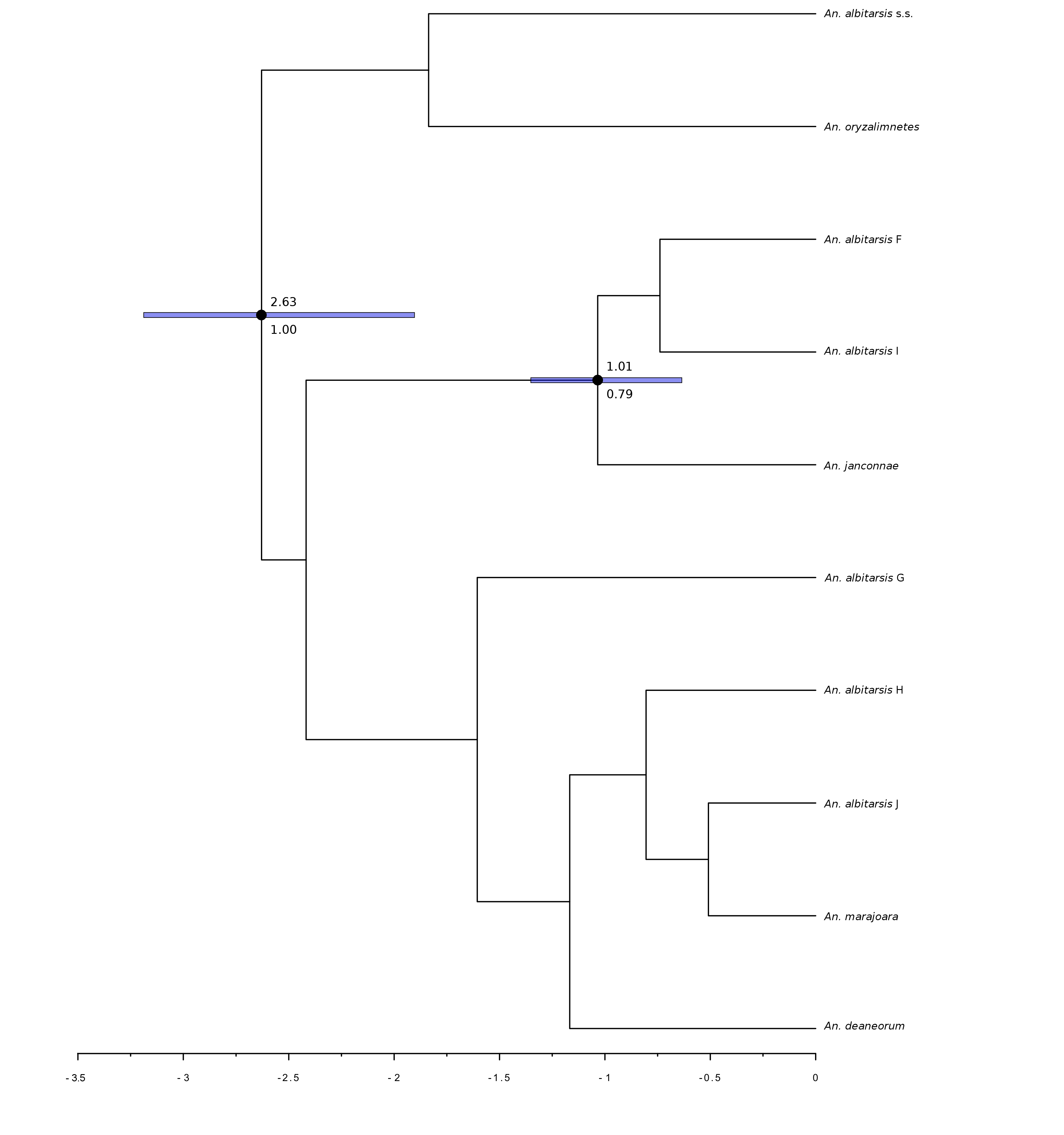

Supplement: Supplementary file 10 — Additional file 10: Figure S6. Species tree of the 13 PCGs of the Albitarsis Complex under a strict-clock model. [file 13071_2021_5090_MOESM10_ESM.png]

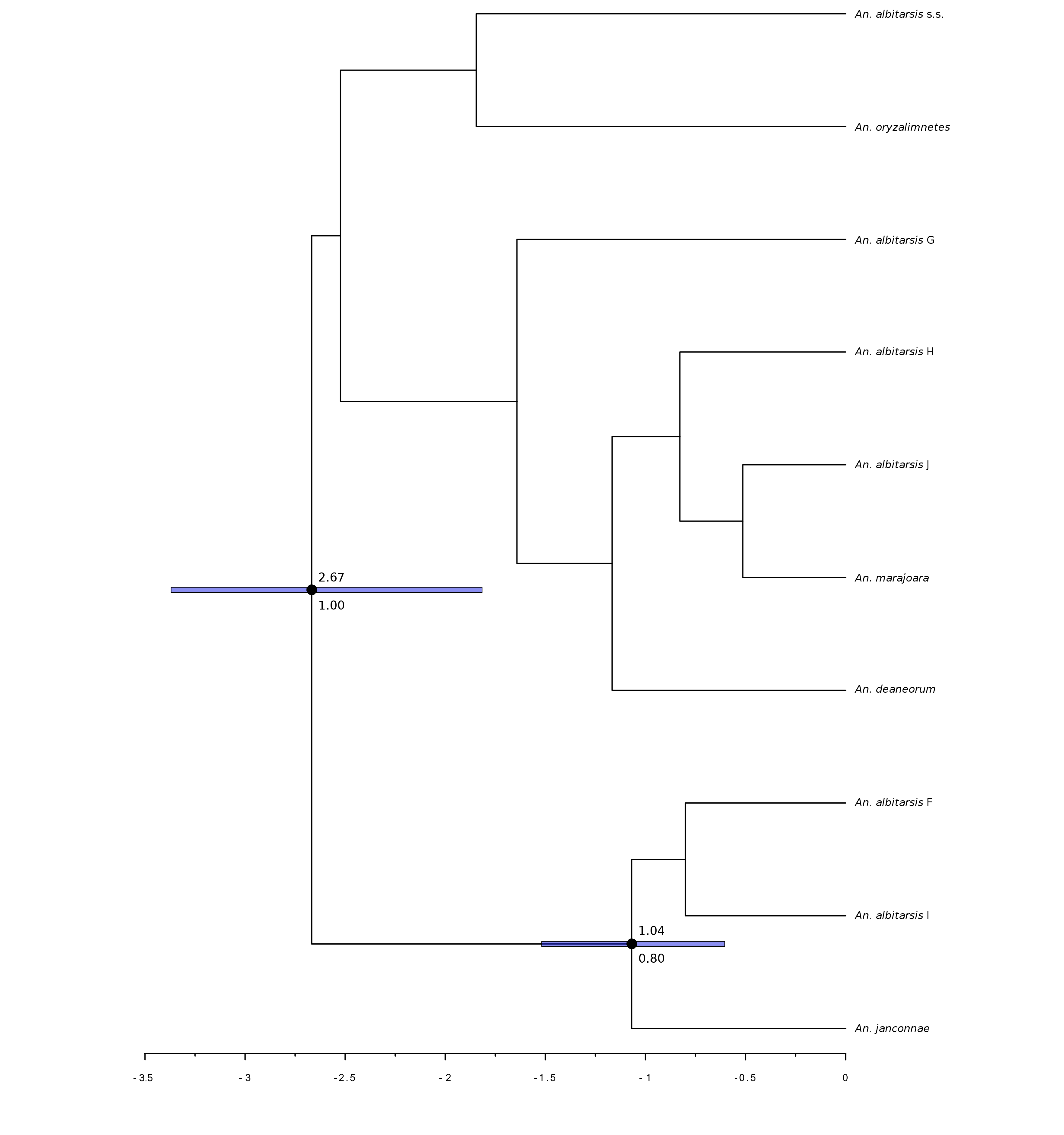

Supplement: Supplementary file 11 — Additional file 11: Figure S7. Species tree of the 13 PCGs of the Albitarsis Complex under a relaxed-clock (UCLN) model. [file 13071_2021_5090_MOESM11_ESM.png]
